# Vitamin D deficiency and the long-term risk of new-onset abdominal aortic aneurysm and severe aortic events: a multicenter propensity score–matched cohort study

**DOI:** 10.3389/fnut.2026.1900016

**Published:** 2026-09-03

**Authors:** Kuo-Chuan Hung, Ting-Sian Yu, I-Wen Chen

**Affiliations:** 1 Department of Anesthesiology, Chi Mei Medical Center, Tainan, Taiwan; 2 Department of Anesthesiology, E-Da Hospital, I-Shou University, Kaohsiung, Taiwan; 3 Department of Anesthesiology, Chi Mei Hospital, Liouying, Tainan, Taiwan

**Keywords:** abdominal aortic aneurysm, aortic dissection, electronic health records, propensity score matching, TriNetX, vitamin D deficiency

## Abstract

**Background:**

Vitamin D deficiency (VDD) is prevalent among older adults and may influence vascular inflammation, extracellular matrix remodeling, and aortic wall integrity; however, its association with incident abdominal aortic aneurysm (AAA) remains uncertain.

**Methods:**

This multicenter retrospective propensity score–matched cohort study used the TriNetX Global Collaborative Network to evaluate the association between vitamin D deficiency (VDD; serum 25-hydroxyvitamin D < 20 ng/mL) and the long-term risk of incident AAA in adults aged ≥50 years, compared with normal vitamin D status (≥30 ng/mL). A 1-year landmark design was applied, with follow-up extending up to 10 years after the index date. The primary outcome was incident AAA. The exploratory secondary outcomes included ruptured AAA, aortic dissection, thoracic aortic aneurysm, non-aortic aneurysm, and all-cause mortality.

**Results:**

After propensity score matching, 466,332 patients were included in each cohort. VDD was associated with a higher risk of incident AAA than normal vitamin D status (0.66% vs. 0.45%; hazard ratio [HR] 1.40, 95% confidence interval [CI], 1.33–1.48; *p* < 0.001). Among the exploratory secondary outcomes, VDD showed higher nominal hazards of ruptured AAA (HR 3.06, 95% CI 2.05–4.58; *p* < 0.001), aortic dissection (HR 1.34, 95% CI 1.15–1.56; *p* < 0.001), thoracic aortic aneurysm (HR 1.13, 95% CI 1.08–1.20; *p* < 0.001), and all-cause mortality (HR 1.48, 95% CI 1.46–1.50; *p* < 0.001), whereas non-aortic aneurysms showed a weaker near-null association (HR 1.05, 95% CI 1.00–1.10; *p* = 0.053). This association persisted across sensitivity analyses and multivariable adjustments.

**Conclusion:**

Vitamin D deficiency was associated with an increased long-term risk of incident AAA, whereas the association with ruptured AAA was exploratory and should be interpreted cautiously because of the small number of events. Given the observational design and modest absolute risk difference, prospective studies are needed to determine whether this association is causal or clinically actionable.

## 1 Introduction

Abdominal aortic aneurysm (AAA) is a clinically silent but potentially fatal vascular disease that may progress to rupture, emergency surgery, and death (1–4). Its pathogenesis is characterized by chronic vascular inflammation, extracellular matrix degradation, vascular smooth muscle cell dysfunction, elastin fragmentation, and progressive loss of aortic wall integrity (5–9). Although advanced age, male sex, smoking, hypertension, and atherosclerotic disease are well-established risk factors (10–12), additional potentially modifiable risk markers related to aortic wall degeneration remain undefined.

Vitamin D deficiency (VDD) is common in older adults and patients with cardiometabolic comorbidities (13, 14). Beyond its classical role in calcium and bone metabolism, vitamin D signaling may influence vascular inflammation, oxidative stress, endothelial function, vascular smooth muscle cell phenotype, and extracellular matrix remodeling (15–18). These pathways are directly relevant to aneurysm biology because AAA formation involves inflammatory cell infiltration, matrix metalloproteinase activation, medial degeneration, and weakening of the aortic wall (19–21). Experimental evidence (22) further suggests that VDD may promote larger and more rupture-prone AAA phenotypes, potentially through enhanced extracellular matrix remodeling and upregulation of osteopontin and matrix metalloproteinases, thereby providing a biologically plausible link between low vitamin D status and impaired aortic wall stability in patients with VDD.

However, human epidemiological evidence remains limited and inconsistent. A recent UK Biobank cohort study reported an inverse association between serum 25-hydroxyvitamin D concentrations and incident AAA (23), whereas the ARIC prospective cohort found little evidence that vitamin D-related biomarkers were associated with incident AAA (24). Moreover, previous studies have focused mainly on AAA occurrence (23, 24), leaving uncertainty regarding whether clinically defined VDD is associated with severe aortic outcomes, including AAA rupture and aortic dissection. Therefore, we conducted a multicenter propensity score–matched cohort study using a large real-world electronic health record network to evaluate the association between VDD and the long-term risk of incident AAA. We also examined severe aortic events, including ruptured AAA and aortic dissection, as secondary outcomes. We hypothesized that patients with VDD would have a higher long-term risk of incident AAA and severe aortic events than those with normal vitamin D status.

## 2 Materials and methods

### 2.1 Study design and data source

This retrospective propensity score–matched cohort study was conducted using the TriNetX Global Collaborative Network, a federated research platform that aggregates longitudinal, de-identified electronic health records from multiple participating healthcare organizations. The TriNetX network has been widely used in peer-reviewed epidemiological and clinical outcomes research, supporting its utility for large-scale real-world evidence studies (25–28). Because the dataset contained no directly identifiable patient information, the need for individual informed consent was waived. The study protocol was approved by the Institutional Review Board of Chi Mei Medical Center (Tainan, Taiwan) and was conducted in accordance with the Declaration of Helsinki and relevant institutional guidelines.

### 2.2 Study population and exposure classification

The study enrolled adults aged ≥50 years with at least one documented serum 25-hydroxyvitamin D [25(OH)D] measurement recorded between January 1, 2010, and December 31, 2023. To confirm ongoing engagement with the healthcare system and allow reliable ascertainment of baseline comorbidities, each individual was required to have a minimum of two healthcare encounters within the preceding 5-year window.

Participants were stratified according to their initial qualifying 25(OH)D values. Those with a concentration below 20 ng/mL were assigned to the VDD cohort, whereas individuals with a value of 30 ng/mL or higher formed the control cohort. The date of the qualifying measurement was used as the index date. To strengthen exposure specificity, individuals in the VDD cohort who had any recorded 25(OH)D value at or above 20 ng/mL within the preceding 3 years were removed, and control participants with any value below 30 ng/mL during the same look-back window were excluded. This restriction was intended to identify consistently documented VDD and reduce misclassification from transiently low 25(OH)D values. This approach better aligned with the hypothesized chronic vascular effects of VDD, although potential selection related to frailty, chronic illness, or greater clinical monitoring cannot be fully excluded.

### 2.3 Exclusion criteria

Individuals with a pre-existing diagnosis of aortic aneurysm or aortic dissection before the index date were excluded to ensure an aortic disease–free baseline. Conditions known to markedly alter vitamin D metabolism, including end-stage renal disease, stage 4 or 5 chronic kidney disease, renal dialysis dependence, prior bariatric surgery, hepatic failure, Crohn’s disease, and celiac disease, also warranted exclusion. To reduce confounding from acute illness–driven vitamin D testing or transient fluctuations in vitamin D levels, individuals who experienced acute kidney failure, sepsis, severe sepsis, critical care utilization, or hospital admission within 30 days before the index date were excluded. Finally, patients with hereditary, congenital, inflammatory, or infectious conditions associated with aortopathy, namely Marfan syndrome, bicuspid aortic valve, Ehlers–Danlos syndrome, Takayasu arteritis, systemic connective tissue disorders, syphilis, and human immunodeficiency virus infection, were excluded. The diagnostic codes for all exclusion criteria are detailed in Supplementary Table 1.

### 2.4 Landmark design

To mitigate reverse causation and immortal-time bias, a 1-year landmark period was imposed. Although the index date corresponded to the qualifying 25(OH)D measurement, follow-up for outcome events commenced only 365 days later and extended up to 10 years from the index date. Patients diagnosed with aortic aneurysm or aortic dissection or who died during this landmark interval were excluded from the analytic cohort.

### 2.5 Propensity score matching

Cohort comparability was optimized through 1:1 propensity score matching using a greedy nearest-neighbor algorithm with a caliper width of 0.1 pooled standard deviation. The propensity model incorporated prespecified baseline covariates encompassing demographic factors, established AAA risk factors, cardiometabolic and vascular comorbidities, renal and hepatic conditions, and relevant medication. As TriNetX operates as a federated analytics platform, investigators do not have access to raw patient-level data to perform multiple imputation. Consequently, structured laboratory and measurement variables were summarized based on their availability, while diagnosis-based covariates were treated as documented EHR indicators within the 5-year baseline period. The absence of a diagnostic code was interpreted as the absence of a documented condition, although some degree of under-ascertainment cannot be excluded.

Laboratory values (hemoglobin, hemoglobin A1c, albumin, and estimated glomerular filtration rate) were not included in the primary matching model because these parameters have variable completeness in electronic health record databases, and the TriNetX platform does not support imputation of missing values. More importantly, laboratory availability in EHR data is unlikely to be random and may reflect healthcare utilization, comorbidity burden, disease severity, and monitoring intensity. Requiring complete laboratory data for the primary matching model could therefore restrict the analysis to a more intensively monitored subgroup and introduce selection bias. Balance was appraised using standardized mean differences, with a threshold of <0.10 deemed satisfactory. Propensity score density distributions were visually compared before and after matching to confirm adequate overlap between the groups. The diagnostic codes for all matching variables are summarized in Supplementary Table 2.

### 2.6 Outcomes

The primary endpoint was incident AAA, including both ruptured and non-ruptured AAA. The secondary endpoints included ruptured AAA, thoracic aortic aneurysm, aortic dissection, non-aortic aneurysm, and all-cause mortality. Non-aortic aneurysms included cerebral, peripheral arterial, and visceral arterial aneurysms, which were examined to assess the anatomical specificity of the observed association. A substantially weaker association with non-aortic aneurysms would support the interpretation that VDD is more closely related to aortic-specific pathobiology than to a generalized predisposition to aneurysms. Secondary hyperparathyroidism, a recognized consequence of VDD, was evaluated as a positive control outcome to support the biological validity of the exposure definition, whereas appendicitis was used as a negative-control outcome. The diagnostic codes for all outcomes are provided in Supplementary Table 3.

### 2.7 Sensitivity, subgroup, and multivariable analyses

Four sensitivity models were used to evaluate the consistency of the primary findings. Model I was confined to patients who survived the entire observation period to assess whether the differential mortality could account for the observed association. Model II required at least one documented healthcare encounter during follow-up, thereby restricting the analysis to individuals with confirmed ongoing clinical observation. Model III was limited to individuals who underwent abdominal computed tomography or ultrasonography during follow-up, addressing potential detection bias, whereby AAA diagnoses may be more frequent in patients receiving more intensive evaluation. Model IV additionally incorporated laboratory parameters (hemoglobin, hemoglobin A1c, albumin, and estimated glomerular filtration rate) into the propensity score model to assess whether the primary findings persisted after balancing these laboratory markers.

Subgroup analyses stratified by sex and age (50–65 vs. >65 years) were performed using interaction terms to evaluate potential effect modification. A multivariable Cox proportional hazards model was additionally fitted, simultaneously adjusting for established AAA risk factors, to assess the consistency of the association between VDD and incident AAA and to verify that the model reproduced the expected direction and magnitude of known risk factors for internal validity.

### 2.8 Exposure-gradient analysis

To examine a potential exposure–gradient relationship, a supplementary analysis was performed comparing patients with vitamin D insufficiency (25(OH)D 20–30 ng/mL) with controls with normal vitamin D status (≥30 ng/mL). The propensity score matching variables and analytical approach were identical to those used in the primary analyses.

### 2.9 Statistical methods

Time-to-event data were modeled using Cox proportional hazards regression, yielding hazard ratios (HRs) with 95% confidence intervals (CIs). A cause-specific hazard framework was adopted for the primary analysis, as the aim was to quantify the association between VDD and AAA occurrence rather than estimating the absolute cumulative incidence. Because the TriNetX analytics platform does not currently permit Aalen–Johansen competing-risk estimation within propensity score–matched cohorts, the Aalen–Johansen estimator was applied only as a supplemental descriptive analysis in the full unmatched cohort, with all-cause mortality treated as a competing event for incident AAA. These estimates were obtained separately for each cohort and were not interpreted as a confounding-adjusted between-group comparison. The proportional hazards assumption was assessed using Schoenfeld residual analysis. To gauge vulnerability to unmeasured confounding, *E*-values were computed for the primary outcome. The *E*-value represents the minimum strength of association that an unmeasured confounder would need to have with both the exposure and the outcome, above and beyond measured covariates, to fully explain the observed HR. Statistical significance for the primary outcome was defined as a two-sided α of 0.05. Formal statistical inference was restricted to the primary outcome. Analyses of secondary endpoints, control outcomes, sensitivity models, exposure-gradient analyses, and subgroup comparisons were considered exploratory and hypothesis-generating. Therefore, *p*-values for these non-primary analyses were reported as nominal descriptive measures only, and no multiplicity correction was applied. These *p*-values should not be interpreted as confirmatory evidence of independent associations. Missing data were handled based on the available information within the database, with no imputation performed.

## 3 Results

### 3.1 Patient selection, follow-up duration, and baseline characteristics

**FIGURE 1:**
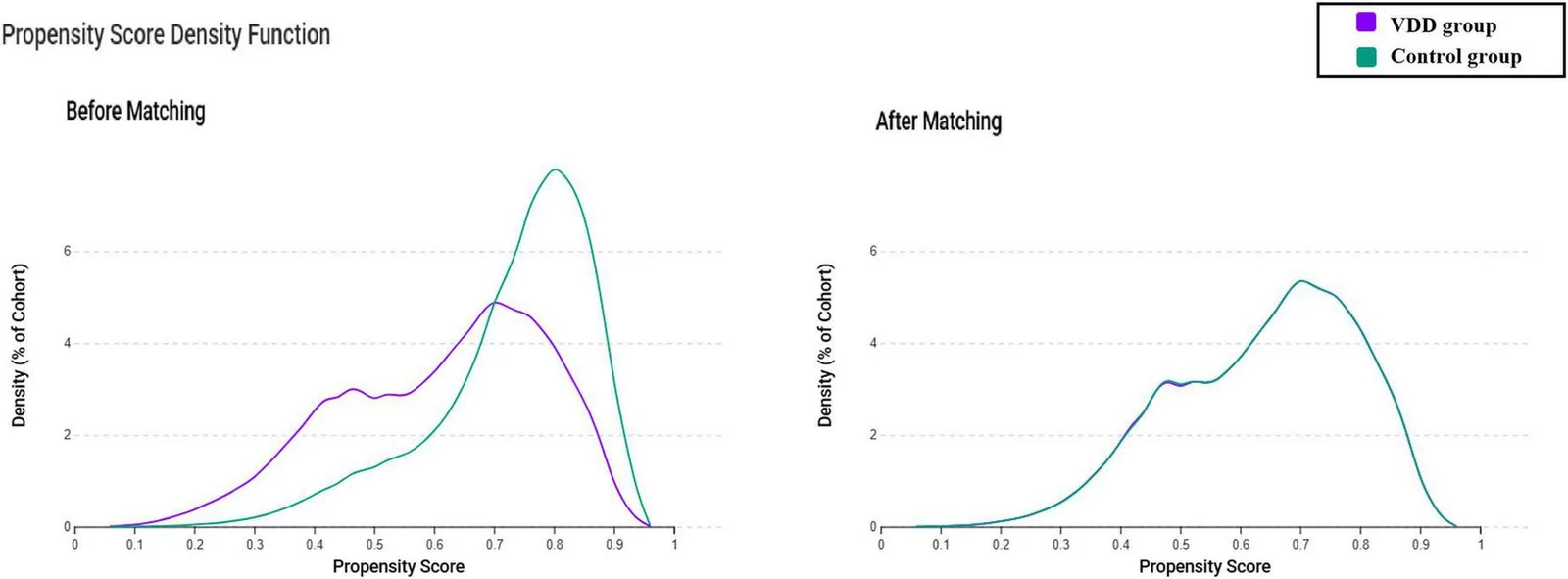
Propensity score density distributions before and after matching. Propensity score density distributions are shown for patients with vitamin D deficiency and controls with normal vitamin D status before and after 1:1 propensity score matching. Improved overlap after matching indicates better covariate balance between the two cohorts. Vitamin D deficiency was defined as serum 25(OH)D < 20 ng/mL, and normal vitamin D status was defined as serum 25(OH)D ≥ 30 ng/mL.

Before propensity score matching, 510,334 patients met the criteria for VDD, and 1,232,018 patients were classified as controls with normal vitamin D status. During the 365-day landmark period, 20,115 patients in the VDD cohort and 45,518 patients in the control cohort were excluded because they met the landmark exclusion criteria. After applying the exclusion criteria and 1:1 propensity score matching, 466,332 patients were included in each cohort. Following matching, all standardized mean differences were below 0.10, indicating a satisfactory covariate balance across demographic characteristics, comorbidities, healthcare utilization variables, and medication use (Table 1). Propensity score density plots also demonstrated improved overlap between the cohorts after matching (Figure 1). The mean follow-up duration was 6.01 ± 3.00 years in the VDD cohort and 5.77 ± 2.91 years in the control group. The median follow-up duration was 5.98 years in the VDD cohort and 5.45 years in the control cohort, with corresponding interquartile ranges of 5.54 and 5.10 years.

**TABLE 1:** Baseline characteristics of patients with low vitamin D status and matched controls before and after propensity score matching.

Variables	Before matching			After matching		
	VDD group (*n* = 510,334)	Control group (*n* = 1,232,018)	SMD	VDD group (*n* = 466,332)	Control group (*n* = 466,332)	SMD
Patient characteristics						
Age at index (years)	59.8 ± 12.1	64.1 ± 11.6	0.367	60.5 ± 12.2	61.0 ± 11.5	0.038
BMI ≥ 30 (kg/m^2^)	189078 (37.1)	343109 (27.8)	0.197	162372 (34.8)	168763 (36.2)	0.029
Female	343412 (67.3)	899176 (73.0)	0.125	318216 (68.2)	316001 (67.8)	0.010
White	282776 (55.4)	964601 (78.3)	0.501	280423 (60.1)	275175 (59.0)	0.023
Black or African American	109770 (21.5)	103066 (8.4)	0.375	80703 (17.3)	80589 (17.3)	0.001
Asian	17496 (3.4)	44991 (3.7)	0.012	17311 (3.7)	17366 (3.7)	0.001
Comorbidities and healthcare utilization						
Essential (primary) hypertension	221166 (43.3)	520294 (42.2)	0.022	196059 (42.0)	200208 (42.9)	0.018
Dyslipidemia	201600 (39.5)	561700 (45.6)	0.123	185793 (39.8)	191169 (41.0)	0.023
Encounter for general adult medical examination	155447 (30.5)	432608 (35.1)	0.099	143713 (30.8)	144114 (30.9)	0.002
Neoplasms	128474 (25.2)	363885 (29.5)	0.098	119076 (25.5)	118598 (25.4)	0.002
Overweight and obesity	110174 (21.6)	180261 (14.6)	0.181	91690 (19.7)	93797 (20.1)	0.011
Diabetes mellitus	101044 (19.8)	188192 (15.3)	0.119	86732 (18.6)	88387 (19.0)	0.009
Disorders of thyroid gland	76131 (14.9)	253035 (20.5)	0.148	72271 (15.5)	73143 (15.7)	0.005
Nicotine dependence	62334 (12.2)	78009 (6.3)	0.204	48433 (10.4)	48392 (10.4)	0.000
Ischemic heart diseases	51613 (10.1)	122496 (9.9)	0.006	45979 (9.9)	46245 (9.9)	0.002
Diseases of liver	32113 (6.3)	64059 (5.2)	0.047	28160 (6.0)	28084 (6.0)	0.001
Other chronic obstructive pulmonary disease	32831 (6.4)	59221 (4.8)	0.071	27895 (6.0)	27837 (6.0)	0.001
Chronic kidney disease (CKD)	30521 (6.0)	79091 (6.4)	0.018	27440 (5.9)	27963 (6.0)	0.005
Cerebrovascular diseases	29108 (5.7)	69689 (5.7)	0.002	25676 (5.5)	25997 (5.6)	0.003
Iron deficiency anemia	26330 (5.2)	50802 (4.1)	0.049	22103 (4.7)	22189 (4.8)	0.001
Heart failure	23527 (4.6)	43375 (3.5)	0.055	19387 (4.2)	19354 (4.2)	0.000
Peripheral vascular diseases	15420 (3.0)	36457 (3.0)	0.004	13667 (2.9)	13518 (2.9)	0.002
Alcohol related disorders	16621 (3.3)	22445 (1.8)	0.091	12626 (2.7)	12510 (2.7)	0.002
COVID-19	12934 (2.5)	34610 (2.8)	0.017	12016 (2.6)	11934 (2.6)	0.001
Malnutrition	4048 (0.8)	8512 (0.7)	0.012	3508 (0.8)	3524 (0.8)	0.000
Medications						
Glucocorticoids	184146 (36.1)	452424 (36.7)	0.013	165636 (35.5)	166231 (35.6)	0.003
Antilipemic agents	141035 (27.6)	370032 (30.0)	0.053	127784 (27.4)	130640 (28.0)	0.014
Beta blockers/related	108933 (21.3)	256833 (20.8)	0.012	96469 (20.7)	97726 (21.0)	0.007
ACE inhibitors	97000 (19.0)	190668 (15.5)	0.094	83025 (17.8)	83403 (17.9)	0.002
Calcium channel blockers	88015 (17.2)	183927 (14.9)	0.063	75139 (16.1)	76008 (16.3)	0.005
Angiotensin II inhibitor	55008 (10.8)	146624 (11.9)	0.035	50122 (10.7)	50924 (10.9)	0.006
Vitamin D supplementation	47976 (9.4)	192693 (15.6)	0.189	46351 (9.9)	48821 (10.5)	0.017
Insulins and analogues	50551 (9.9)	78051 (6.3)	0.131	41197 (8.8)	41229 (8.8)	0.000

Data are presented as number (%) or mean ± standard deviation. Standardized mean differences <0.10 were considered acceptable for covariate balance. ACE, angiotensin-converting enzyme; BMI, body mass index; CKD, chronic kidney disease; SMD, standardized mean difference; VDD, vitamin D deficiency.

### 3.2 Primary, secondary, and control outcomes

**FIGURE 2:**
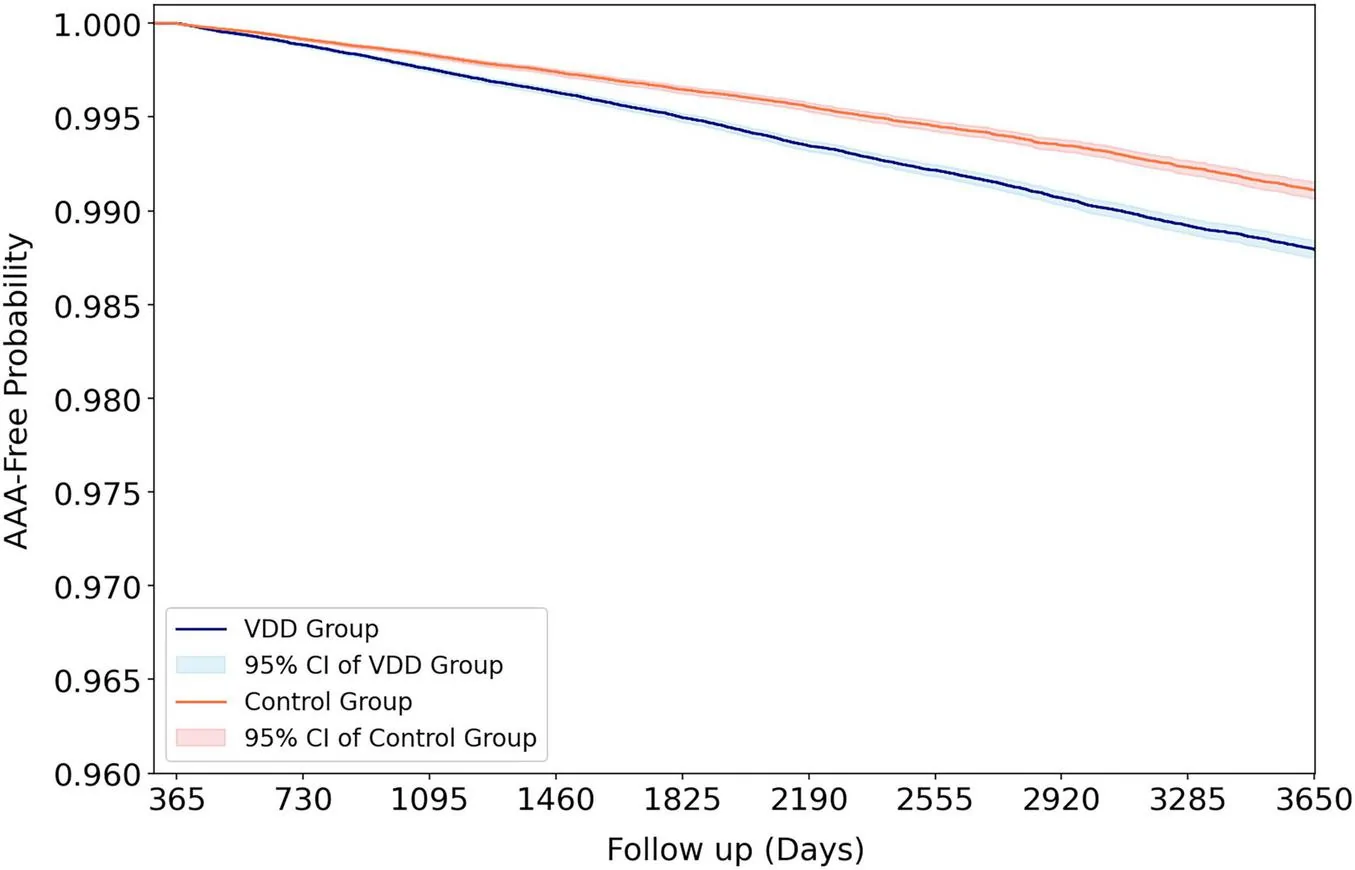
Kaplan–Meier curves for abdominal aortic aneurysm–free probability. Kaplan–Meier curves show abdominal aortic aneurysm (AAA)–free probability over the 10-year follow-up period in the propensity score–matched cohort. Follow-up began after the 1-year landmark period. Patients with vitamin D deficiency had a lower AAA–free probability than controls with normal vitamin D status. Shaded areas represent 95% confidence intervals. Vitamin D deficiency was defined as serum 25(OH)D < 20 ng/mL, and normal vitamin D status was defined as serum 25(OH)D ≥ 30 ng/mL. AAA, abdominal aortic aneurysm; VDD, vitamin D deficiency.

During the 10-year observation window, VDD was associated with a higher risk of incident AAA than normal vitamin D status (3,086 [0.66%] vs. 2,087 [0.45%]; HR 1.40, 95% CI 1.33–1.48; *p* < 0.001; Figure 2 and Table 2). The absolute event rate difference was 0.21 percentage points. The *E*-value was 2.15 for the point estimate and 1.99 for the lower bound of the 95% CI.

**TABLE 2:** Association between vitamin D deficiency and 10-year risks of abdominal aortic aneurysm.

Outcome	VDD group (*n* = 466,332)	Control group (*n* = 466,332)	HR (95% CI)	*P*-value
	Events (%)	Events (%)		
Primary outcome				
AAA	3,086 (0.66)	2,087 (0.45)	1.40 (1.33–1.48)	<0.001
Secondary outcomes				
AAA rupture	102 (0.02)	31 (0.01)	3.06 (2.05–4.58)	<0.001
TAA	2,990 (0.64)	2,488 (0.53)	1.13 (1.08–1.20)	<0.001
Aortic dissection	402 (0.09)	284 (0.06)	1.34 (1.15–1.56)	<0.001
Non-aortic aneurysm	3,825 (0.82)	3,493 (0.75)	1.05 (1.00–1.10)	0.053
Mortality	39,942 (8.57)	25,782 (5.53)	1.48 (1.46–1.50)	<0.001
Positive control outcome				
Secondary hyperparathyroidism	1,147 (0.25)	641 (0.14)	1.72 (1.56–1.89)	<0.001
Negative control outcome				
Appendicitis	2,339 (0.50)	2,096 (0.45)	1.06 (1.00–1.13)	0.046

Data are presented as number of events (%). AAA, abdominal aortic aneurysm; CI, confidence interval; HR, hazard ratio; TAA, thoracic aortic aneurysm; VDD, vitamin D deficiency.

Among the secondary outcomes, VDD was associated with ruptured AAA (HR 3.06, *p* < 0.001), aortic dissection (HR 1.34, *p* < 0.001), thoracic aortic aneurysm (HR 1.13, *p* < 0.001), and all-cause mortality (HR 1.48, *p* < 0.001). The association with non-aortic aneurysms was weaker and did not reach statistical significance (HR 1.05, *p* = 0.053). For control outcomes, VDD was associated with secondary hyperparathyroidism (HR 1.72, *p* < 0.001), whereas appendicitis showed only a small borderline association (HR 1.06, *p* = 0.046).

### 3.3 Sensitivity and subgroup analyses

The association between VDD and incident AAA remained consistent across all sensitivity models, with HRs ranging from 1.36–1.52 (Table 3). Similar patterns were observed for ruptured AAA and aortic dissection in the sensitivity analyses. In the imaging-restricted model, which included only patients who underwent at least one abdominal computed tomography or ultrasonography examination during follow-up, the association between VDD and incident AAA remained, suggesting that the primary finding was not entirely explained by differences in abdominal imaging ascertainment. In the laboratory-adjusted matching model, VDD also remained associated with incident AAA after additional balancing of hemoglobin, hemoglobin A1c, albumin, and estimated glomerular filtration rate. Before laboratory-adjusted matching, missingness differed across structured laboratory and measurement variables (Supplementary Table 4). The missing proportions in the VDD and control cohorts were 19.3% and 27.1% for estimated glomerular filtration rate (eGFR), 23.2% and 33.1% for hemoglobin, 34.2% and 39.6% for albumin, 39.2% and 35.4% for body mass index, and 58.2% and 66.8% for hemoglobin A1c, respectively. These data show that laboratory and measurement completeness varied across variables, supporting the decision to evaluate these markers in a sensitivity analysis rather than in the primary matching model.

**TABLE 3:** Sensitivity analyses for the association between vitamin D deficiency and 10-year risks of abdominal aortic aneurysm and related aortic outcomes.

Outcomes	Model I		Model II		Model III		Model IV	
	HR (95% CI)	*P*-value	HR (95% CI)	*P*-value	HR (95% CI)	*P*-value	HR (95% CI)	*P*-value
AAA	1.36 (1.27–1.44)	<0.001	1.42 (1.34–1.50)	<0.001	1.52 (1.42–1.63)	<0.001	1.40 (1.33–1.48)	<0.001
AAA rupture	2.24 (1.44–3.48)	<0.001	2.45 (1.70–3.54)	<0.001	2.45 (1.60–3.73)	<0.001	2.30 (1.61–3.29)	<0.001
TAA	1.09 (1.03–1.16)	0.002	1.13 (1.07–1.19)	<0.001	1.27 (1.18–1.36)	<0.001	1.13 (1.07–1.19)	<0.001
Aortic dissection	1.40 (1.17–1.68)	<0.001	1.37 (1.18–1.60)	<0.001	1.29 (1.06–1.56)	0.012	1.23 (1.06–1.43)	0.006
Non-aortic aneurysm	0.99 (0.94–1.04)	0.714	1.04 (0.99–1.09)	0.097	1.10 (1.04–1.17)	0.001	1.05 (1.01–1.10)	0.030
Mortality	NA	NA	1.47 (1.44–1.49)	<0.001	1.42 (1.39–1.46)	<0.001	1.48 (1.46–1.51)	<0.001

Model I was restricted to patients who survived throughout follow-up (*n* = 425,660 for each group). Model II was restricted to patients with at least one recorded healthcare encounter during follow-up (*n* = 445,290 for each group). Model III was restricted to patients with at least one recorded abdominal computed tomography or ultrasonography examination during follow-up (*n* = 120,142 for each group). Model IV additionally included hemoglobin, hemoglobin A1c, albumin, and estimated glomerular filtration rate in propensity score matching (*n* = 461,521 for each group). AAA, abdominal aortic aneurysm; CI, confidence interval; CT, computed tomography; eGFR, estimated glomerular filtration rate; HR, hazard ratio; NA, not available; TAA, thoracic aortic aneurysm; VDD, vitamin D deficiency.

In sex-stratified analyses, VDD was associated with incident AAA in both males (HR 1.45, *p* < 0.001) and females (HR 1.61, *p* < 0.001), without a significant interaction by sex (*p* for interaction = 0.080; Table 4). Age-stratified analyses showed similar associations in patients aged 50–65 years (HR 1.30, *p* = 0.001) and those older than 65 years (HR 1.49, *p* < 0.001), without significant interaction by age group (*p* for interaction = 0.103; Table 5).

**TABLE 4:** Sex-stratified subgroup analyses for the association between vitamin D deficiency and 10-year risks of abdominal aortic aneurysm.

Outcomes	Male (*n* = 147,947 for each group)		Female (*n* = 317,309 for each group)		P for interaction
	HR (95% CI)	*P*-value	HR (95% CI)	*P*-value	
AAA	1.45 (1.34–1.55)	<0.001	1.61 (1.47–1.76)	<0.001	0.080
AAA rupture	2.96 (1.79–4.90)	<0.001	1.66 (1.00–2.73)	0.046	0.152
TAA	1.09 (1.01–1.17)	0.022	1.30 (1.20–1.41)	<0.001	0.002
Aortic dissection	1.18 (0.95–1.48)	0.142	1.57 (1.27–1.94)	<0.001	0.074
Non-aortic aneurysm	1.14 (1.06–1.23)	0.001	0.97 (0.92–1.03)	0.314	0.001
Mortality	1.50 (1.47–1.54)	<0.001	1.47 (1.44–1.50)	<0.001	0.202

AAA, abdominal aortic aneurysm; CI, confidence interval; HR, hazard ratio; TAA, thoracic aortic aneurysm; VDD, vitamin D deficiency.

**TABLE 5:** Age-stratified subgroup analyses for the association between vitamin D deficiency and 10-year risks of abdominal aortic aneurysm.

Outcomes	50–65 years (*n* = 200,926 for each group)		>65 years (*n* = 264,850 for each group)		*P* for interaction
	HR (95% CI)	*P*-value	HR (95% CI)	*P*-value	
AAA	1.30 (1.11–1.53)	0.001	1.49 (1.40–1.58)	<0.001	0.103
AAA rupture	NA	NA	3.18 (2.08–4.84)	<0.001	NA
TAA	1.06 (0.95–1.18)	0.329	1.21 (1.13–1.28)	<0.001	0.032
Aortic dissection	1.29 (0.93–1.78)	0.131	1.39 (1.17–1.65)	<0.001	0.688
Non-aortic aneurysm	1.10 (1.00–1.20)	0.051	1.04 (0.99–1.10)	0.113	0.303
Mortality	1.43 (1.37–1.49)	<0.001	1.47 (1.45–1.50)	<0.001	0.228

AAA, abdominal aortic aneurysm; CI, confidence interval; HR, hazard ratio; NA, not available; TAA, thoracic aortic aneurysm; VDD, vitamin D deficiency.

### 3.4 Multivariable Cox regression, competing-risk analysis, and exposure-gradient analysis

In the multivariable Cox model fitted to the unmatched cohort, VDD remained associated with a higher risk of incident AAA after adjustment for demographic, cardiometabolic, vascular, and clinical risk factors (HR 1.52, *p* < 0.001; Table 6). The model reproduced several expected associations with AAA, including higher risks associated with male sex, older age, nicotine dependence, chronic obstructive pulmonary disease, ischemic heart disease, peripheral vascular disease, and hypertension. Diabetes mellitus was inversely associated with incident AAA (HR 0.78, *p* < 0.001), consistent with prior epidemiological observations showing a lower AAA risk among patients with diabetes.

**TABLE 6:** Multivariable Cox regression model for incident abdominal aortic aneurysm in the unmatched cohort.

Variables	HR (95% CI)	*P*-value
VDD vs. control group	1.52 (1.45–1.59)	<0.001
Male	3.33 (3.19–3.47)	<0.001
Age at index	1.06 (1.06–1.06)	<0.001
Dyslipidemia	1.10 (1.04–1.15)	<0.001
Overweight and obesity	1.00 (0.94–1.06)	0.913
Diabetes mellitus	0.78 (0.74–0.82)	<0.001
Nicotine dependence	3.12 (2.94–3.30)	<0.001
Ischemic heart diseases	1.54 (1.45–1.62)	<0.001
COPD	1.72 (1.61–1.83)	<0.001
Chronic kidney disease (CKD)	1.00 (0.93–1.07)	0.916
Cerebrovascular diseases	1.19 (1.11–1.27)	<0.001
Heart failure	0.97 (0.89–1.05)	0.427
Peripheral vascular diseases	1.41 (1.31–1.53)	<0.001
Alcohol-related disorders	0.82 (0.73–0.92)	0.001
Malnutrition	0.93 (0.76–1.13)	0.456
Essential (primary) hypertension	1.30 (1.23–1.37)	<0.001
White	1.35 (1.28–1.42)	<0.001

Hazard ratios were estimated using a multivariable Cox proportional hazards model fitted in the unmatched cohort. CI, confidence interval; CKD, chronic kidney disease; COPD, chronic obstructive pulmonary disease; HR, hazard ratio; VDD, vitamin D deficiency.

As a supplemental descriptive analysis constrained by the TriNetX platform, Aalen–Johansen competing-risk cumulative incidence estimates were generated in the full unmatched cohort, with all-cause mortality treated as a competing event for incident AAA. This analysis showed a cumulative AAA incidence of 1.09% in the VDD cohort and 0.84% in the control cohort (Table 7). The corresponding cumulative mortality estimates were 13.70% and 11.91%, respectively. Because this analysis was performed in the unmatched cohort, it should be interpreted only as an unadjusted descriptive assessment of absolute cumulative incidence and not as a substitute for the primary propensity score–matched cause-specific Cox analysis.

**TABLE 7:** Supplemental descriptive competing-risk cumulative incidence of abdominal aortic aneurysm and all-cause mortality in the full unmatched cohort.

Cohort	Outcome	% of cohort	Aalen–Johansen cumulative incidence at end of time window
VDD	AAA	0.64%	1.09%
VDD	Mortality	8.20%	13.70%
Normal vitamin D status	AAA	0.46%	0.84%
Normal vitamin D status	Mortality	6.45%	11.91%

Cumulative incidence was estimated using the Aalen–Johansen estimator, with all-cause mortality treated as a competing event for incident abdominal aortic aneurysm. AAA, abdominal aortic aneurysm; VDD, vitamin D deficiency.

In the exposure-gradient analysis, vitamin D insufficiency was associated with a higher risk of incident AAA than normal vitamin D status (HR 1.34, *p* < 0.001; Table 8), with a point estimate numerically lower than that observed for VDD in the primary analysis. Similar exposure-gradient patterns were observed for ruptured AAA, aortic dissection, and secondary hyperparathyroidism, whereas appendicitis remained near null.

**TABLE 8:** Exposure-gradient analysis of vitamin D insufficiency and 10-year risks of abdominal aortic aneurysm, severe aortic events, control outcomes, and mortality.

Outcome	VDI group (*n* = 615,798)	Control group (*n* = 615,798)	HR (95% CI)	*P*-value
	Events (%)	Events (%)		
AAA	3,851 (0.63)	2,788 (0.45)	1.34 (1.28–1.41)	<0.001
AAA rupture	94 (0.02)	58 (0.01)	1.56 (1.12–2.16)	0.007
TAA	4,379 (0.71)	3,480 (0.57)	1.22 (1.17–1.28)	<0.001
Aortic dissection	469 (0.08)	377 (0.06)	1.20 (1.05–1.38)	0.007
Non-aortic aneurysm	4,938 (0.80)	4,643 (0.75)	1.04 (1.00–1.08)	0.072
Mortality	43,698 (7.10)	33,249 (5.40)	1.28 (1.26–1.30)	<0.001
Hyperparathyroidism	976 (0.16)	879 (0.14)	1.09 (0.99–1.19)	0.076
Appendicitis	3,052 (0.50)	2,915 (0.47)	1.02 (0.97–1.07)	0.460

Data are presented as number of events (%). Vitamin D insufficiency was defined as serum 25(OH)D 20–30 ng/mL (*n* = 615,798), and normal vitamin D status was defined as serum 25(OH)D ≥ 30 ng/mL (*n* = 615,798). AAA, abdominal aortic aneurysm; CI, confidence interval; HR, hazard ratio; TAA, thoracic aortic aneurysm; VDI, vitamin D insufficiency.

## 4 Discussion

In this large multicenter propensity score–matched cohort study, clinically defined VDD was associated with a higher long-term risk of incident AAA than normal vitamin D status. This association was consistent across sensitivity analyses addressing survival, follow-up ascertainment, imaging detection, and laboratory balance and was further supported by multivariable adjustment in the unmatched cohort. Exploratory analyses also suggested associations with ruptured AAA and aortic dissection, whereas the association with non-aortic aneurysms was comparatively weaker, supporting possible aortic specificity. However, given the modest absolute risk difference and observational design, these findings should be interpreted as hypothesis-generating evidence of association rather than a causal relationship.

The observed association between VDD and incident AAA extends prior epidemiological evidence, which has been limited in scale and inconsistent in direction. The ARIC prospective cohort followed 12,770 participants for a median of 19.7 years and found no significant association between VDD and incident AAA (24), whereas an Australian community-based study of 4,233 older men (29) reported inverse associations between 25(OH)D concentrations and AAA diagnosis or aneurysm size. Recent cardiovascular literature also supports a broader link between vitamin D status and vascular inflammation, endothelial function, oxidative stress, hypertension, and atherosclerosis, although such evidence should be viewed as mechanistic background rather than direct proof of an AAA-specific effect (30). Compared with these earlier studies, the present analysis used a clinically defined VDD threshold, included more than 460,000 matched patients per cohort, and applied a 1-year landmark design to reduce reverse causation bias. In addition, the propensity score model balanced a broad range of clinically relevant AAA risk factors, including smoking-related diagnoses, chronic obstructive pulmonary disease, peripheral vascular disease, chronic kidney disease, cardiovascular comorbidities, healthcare utilization, and the use of medications. These design features may partly explain why our study was able to detect a clinically defined VDD–AAA association that was not consistently observed in smaller cohorts using conventional multivariable adjustments.

Among the exploratory secondary outcomes, VDD was associated with ruptured AAA and aortic dissection. Because these analyses were not adjusted for multiplicity, they should be interpreted strictly as hypothesis-generating and not as confirmatory evidence of independent associations. The higher relative estimate for ruptured AAA is biologically plausible and compatible with experimental evidence (22), linking VDD to aneurysm enlargement and rupture-prone phenotypes. The lower HRs for ruptured AAA in the sensitivity analyses likely reflected rare-event instability and attenuation after restricting the analytic population; therefore, the rupture finding should be interpreted as a hypothesis-generating signal rather than a precise estimate of absolute rupture risk. VDD was also associated with a high risk of thoracic aortic aneurysm, although the magnitude of the association (HR 1.13) was weaker than that observed for AAA. This attenuation is biologically plausible because the pathogenesis of thoracic aortic aneurysm involves distinct mechanisms, including genetic predisposition and cystic medial degeneration (31, 32), which may be less directly influenced by vitamin D–mediated inflammatory and matrix-remodeling pathways.

A notable feature of this study was the inclusion of non-aortic aneurysms as anatomic specificity comparators. In contrast to the stronger association observed for AAA, VDD was not significantly associated with non-aortic aneurysms. This pattern suggests that VDD may be more closely related to aortic-specific degenerative processes than to a generalized aneurysmal predisposition, although this interpretation remains to be confirmed. This interpretation is biologically plausible because AAA pathogenesis is characterized by localized aortic wall inflammation, vascular smooth muscle cell dysfunction, and extracellular matrix degradation. The positive control outcome of secondary hyperparathyroidism supported the biological validity of the definition of VDD exposure. Conversely, the negative-control outcome of appendicitis showed a near-null effect estimate. Because control outcomes were exploratory and no multiplicity correction was applied, the nominal *p*-value for appendicitis was not interpreted as evidence of a meaningful association. Instead, the small effect size suggested that nonspecific residual bias, if present, was limited in magnitude and unlikely to explain the substantially larger primary AAA association (33).

The sensitivity analyses provided methodological support for the primary findings. The association between VDD and incident AAA remained present in models restricted to patients who survived throughout follow-up and those with documented healthcare encounters during follow-up, partially mitigating the concern that the finding was driven solely by differential mortality or incomplete observation. Because AAA is frequently asymptomatic and often detected incidentally during abdominal imaging performed for unrelated indications (34, 35), the imaging-restricted model was particularly relevant for addressing potential ascertainment biases. The association persisted among patients who underwent abdominal computed tomography or ultrasonography during follow-up. The modestly higher HR in this model may reflect enrichment of a clinically higher-risk subgroup and residual differences in imaging indication, timing, or frequency. Because requiring at least one imaging examination did not fully equalize surveillance intensity, this analysis cannot fully eliminate selection or ascertainment bias.

Subgroup analyses suggested that the association between VDD and incident AAA was present in both males and females and in both age strata, without statistically significant interactions with the primary outcome. These findings suggest that the observed association was not confined to any single demographic subgroup. However, the subgroup analyses were exploratory, and the study was not designed to redefine sex- or age-specific screening strategies. The findings in patients aged 50–65 years are clinically interesting because conventional AAA screening strategies primarily focus on older adults, particularly men aged 65 years or older (1). Nevertheless, the absolute risk of AAA in younger individuals is lower, and these findings should not be interpreted as evidence to expand AAA screening eligibility criteria. They suggest that VDD may identify a vascular risk phenotype that warrants further investigation.

The multivariable Cox model fitted to the unmatched cohort provided an additional internal validity check. VDD remained associated with incident AAA after adjusting for demographic, cardiometabolic, vascular, and clinical risk factors. The model also reproduced several expected AAA associations, including higher risks associated with male sex, older age, nicotine dependence, chronic obstructive pulmonary disease, ischemic heart disease, peripheral vascular disease, and hypertension (1, 10, 36). Diabetes mellitus was inversely associated with incident AAA, consistent with prior epidemiological studies and meta-analyses reporting a lower prevalence, incidence, or progression of AAA among patients with diabetes (37–39). Proposed mechanisms include diabetes-related extracellular matrix glycation and collagen cross-linking, reduced matrix metalloproteinase activity, altered inflammatory remodeling of the aortic wall, and potential effects of antidiabetic therapies, all of which may limit aneurysmal dilation despite the broader vascular risk associated with diabetes (37–39). This internal consistency supports the validity of our analytical framework. However, because the multivariable model was performed in the unmatched cohort, it should be considered complementary to, rather than a replacement for, the primary propensity score–matched analysis. The competing risk analysis further showed that the direction of the association persisted when death was considered a competing event; however, this analysis was descriptive and based on the full unmatched cohort. Similarly, the exposure-gradient analysis suggested a graded pattern across vitamin D insufficiency and deficiency, but it should be considered exploratory because the exposure categories were evaluated in separate matched comparisons rather than in a single continuous dose–response model.

Clinical implications should be interpreted cautiously. The present findings do not support VDD as a stand-alone indication for AAA screening, nor do they justify expanding current guideline-based AAA screening criteria. Instead, VDD may be considered a supplementary vascular risk marker in patients who already have established AAA risk factors. In general clinical practice, the presence of VDD may prompt clinicians to review traditional AAA risk factors, prior abdominal imaging, and eligibility for guideline-based screening. From a perioperative perspective, particularly before major noncardiac surgery, VDD in patients with conventional vascular risk factors may highlight the need for careful cardiovascular and vascular risk review and, when aneurysmal disease is known or suspected, multidisciplinary evaluation with vascular specialists. However, vitamin D correction should not be assumed to reduce AAA risk or severe aortic events based on the present observational data.

Several limitations should be acknowledged. First, although both cohorts included only patients with documented 25(OH)D testing, the clinical indication for testing was unavailable and may have differed between groups. Therefore, indication-specific residual confounding cannot be excluded. Furthermore, important factors such as sunlight exposure, dietary intake, physical activity, frailty, socioeconomic status, over-the-counter supplement use, and detailed smoking history were not fully considered. Although race was adequately balanced after matching, the substantial pre-matching racial differences may reflect unmeasured social, geographic, or healthcare-access factors, and residual confounding related to these factors cannot be excluded. Second, although the 3-year look-back was intended to identify a more persistent VDD phenotype, exposure classification remained based on a single qualifying 25(OH)D measurement. Harmonized data on testing season, assay methods, laboratory reference ranges, testing indications, and post-index vitamin D levels or supplementation were unavailable. Therefore, residual exposure misclassification cannot be excluded, and differential vitamin D repletion may have attenuated the observed association over time. Third, incident AAA, ruptured AAA, and aortic dissection were identified using electronic health record diagnosis codes without chart-level adjudication or algorithm validation against imaging-confirmed disease; therefore, outcome misclassification cannot be excluded and could have biased the effect estimates in either direction. Although the imaging-restricted sensitivity analysis partially reduced concerns about differential ascertainment, selection by indication could not be excluded. Fourth, data on aneurysm diameter, growth rate, morphology, rupture presentation, operative repair, imaging findings, and cause-specific mortality were unavailable. In particular, the TriNetX platform does not provide structured diagnostic imaging details, such as baseline aneurysm diameter or serial aortic measurements. Therefore, we could not verify whether small, subclinical aneurysms were already present but unrecorded at the index date. Therefore, we could not evaluate aneurysm severity, longitudinal expansion, treatment pathways, or whether the deaths were directly attributable to aneurysm-related complications. Fifth, secondary outcomes, subgroup analyses, control outcomes, sensitivity analyses, and exposure gradient analyses were exploratory and not adjusted for multiplicity. Therefore, these findings should be interpreted as supportive or hypothesis-generating, rather than as confirmatory evidence. Finally, the requirement for at least two healthcare encounters during the preceding 5 years may have selected more healthcare-engaged and comorbid patients while excluding healthier infrequent users. Accordingly, the findings may not be fully generalizable to community-dwelling populations or individuals with limited healthcare access.

## 5 Conclusion

Clinically defined VDD was associated with a higher long-term risk of incident AAA in this large propensity score–matched cohort study. The association was supported by sensitivity models and multivariable adjustment, whereas secondary outcomes, subgroup analyses, and exposure gradient analyses should be interpreted as exploratory and hypothesis-generating. These findings should not be interpreted as supporting VDD-based AAA screening, but they suggest that VDD may serve as an adjunctive risk marker when evaluating patients with established vascular risk factors in general or perioperative clinical settings. Given the modest absolute risk difference and the observational design, further prospective studies with systematic aortic imaging, repeated vitamin D measurements, detailed lifestyle data, and mechanistic endpoints are needed to determine whether VDD is causally involved in AAA development or severe aortic complications.

## Data Availability

The data supporting the findings of this study were obtained from the TriNetX Global Collaborative Network. Restrictions apply to the availability of these data, which were used under license for the current study and are not publicly accessible. Data access can be obtained through a direct agreement with TriNetX. Requests to access the datasets should be directed to https://live. trinetx.com.
