# Supplementary material for: Vitamin D deficiency and the long-term risk of new-onset abdominal aortic aneurysm and severe aortic events: a multicenter propensity score–matched cohort study
Source: Front Nutr. 2026 Sep 3;13:1900016. doi: 10.3389/fnut.2026.1900016 (PMC13581915; doi:10.3389/fnut.2026.1900016)
Supplement: Supplementary file 1 [file Table_1.docx]

**Supplemental Table 1. Codes used to define exclusion criteria**

| Exclusion category | Condition / clinical factor | Code |
| --- | --- | --- |
| Pre-existing aortic disease | Aortic aneurysm and dissection | I71 |
| Advanced kidney disease | End-stage renal disease | N18.6 |
| Advanced kidney disease | Chronic kidney disease, stage 4 | N18.4 |
| Advanced kidney disease | Chronic kidney disease, stage 5 | N18.5 |
| Dialysis status | Dependence on renal dialysis | Z99.2 |
| Altered vitamin D metabolism / absorption | Bariatric surgery status | Z98.84 |
| Altered vitamin D metabolism / absorption | Hepatic failure, not elsewhere classified | K72 |
| Altered vitamin D metabolism / absorption | Crohn’s disease | K50 |
| Altered vitamin D metabolism / absorption | Celiac disease | K90.0 |
| Recent acute illness | Critical care services | 1013729 |
| Recent acute illness | Acute kidney failure | N17 |
| Recent acute illness | Sepsis | A41 |
| Recent acute illness | Severe sepsis | R65.2 |
| Recent acute illness | Inpatient encounter | IMP |
| Recent acute illness | Inpatient non-acute encounter | NONAC |
| Recent acute illness | Inpatient acute encounter | ACUTE |
| Congenital / inherited aortopathy | Marfan syndrome | Q87.4 |
| Congenital / inherited aortopathy | Bicuspid aortic valve | Q23.81 |
| Congenital / inherited aortopathy | Ehlers–Danlos syndrome | Q79.6 |
| Inflammatory / connective tissue aortopathy | Takayasu arteritis / aortic arch syndrome | M31.4 |
| Inflammatory / connective tissue aortopathy | Systemic connective tissue disorders | M30–M36 |
| Infectious / secondary aortopathy | Congenital syphilis | A50 |
| Infectious / secondary aortopathy | Early syphilis | A51 |
| Infectious / secondary aortopathy | Late syphilis | A52 |
| Infectious / secondary aortopathy | Other and unspecified syphilis | A53 |
| Infectious / secondary aortopathy | Human immunodeficiency virus disease | B20 |
| Infectious / secondary aortopathy | HIV status | 3859 |
| Early mortality exclusion | Deceased | Deceased |
| Early mortality exclusion | Ill-defined and unknown cause of mortality | R99 |

**Supplemental Table 2. Variables and codes used for propensity score matching**

| Category | Variable | Code |
| --- | --- | --- |
| Diagnosis | Neoplasms | C00–D49 |
|  | Overweight and obesity | E66 |
|  | Diabetes mellitus | E08–E13 |
|  | Nicotine dependence | F17 |
|  | Ischemic heart diseases | I20–I25 |
|  | Malnutrition | E40–E46 |
|  | Diseases of liver | K70–K77 |
|  | Disorders of lipoprotein metabolism and other lipidemias | E78 |
|  | Essential hypertension | I10 |
|  | Chronic kidney disease | N18 |
|  | Alcohol-related disorders | F10 |
|  | Chronic obstructive pulmonary disease | J44 |
|  | COVID-19 | U07.1 |
|  | General examination without complaint, suspected, or reported diagnosis | Z00 |
|  | Systemic connective tissue disorders | M30–M36 |
|  | Disorders of thyroid gland | E00–E07 |
|  | Iron deficiency anemia | D50 |
|  | Cerebrovascular diseases | I60–I69 |
|  | Heart failure | I50 |
|  | Other peripheral vascular diseases | I73 |
|  | General adult medical examination | Z00.0 |
| Medication | Vitamin D | VT500 |
|  | Glucocorticoids | H02AB |
|  | Insulins and analogues | A10A |
|  | Beta blockers/related agents | CV100 |
|  | Calcium channel blockers | CV200 |
|  | Antilipemic agents | CV350 |
|  | ACE inhibitors | CV800 |
|  | Angiotensin II inhibitors | CV805 |
| Laboratory | Body mass index | 39156-5 |

**Supplemental Table 3. Codes used to define study outcomes**

| Category | Outcome | Codes / definitions |
| --- | --- | --- |
| Primary outcome | Abdominal aortic aneurysm | I71.3 Abdominal aortic aneurysm, ruptured; I71.4 Abdominal aortic aneurysm, without rupture |
| Secondary outcome | Abdominal aortic aneurysm rupture | I71.3 Abdominal aortic aneurysm, ruptured |
| Secondary outcome | Thoracic aortic aneurysm | I71.1 Thoracic aortic aneurysm, ruptured; I71.2 Thoracic aortic aneurysm, without rupture |
| Secondary outcome | Aortic dissection | I71.0 Dissection of aorta |
| Secondary outcome | Other aneurysm | I72 Other aneurysm;  Aneurysm of carotid artery (I72.0); aneurysm of artery of upper extremity (I72.1); aneurysm of renal artery (I72.2); aneurysm of iliac artery (I72.3); aneurysm of artery of lower extremity (I72.4); aneurysm of other precerebral arteries (I72.5); aneurysm of vertebral artery (I72.6); aneurysm of other specified arteries (I72.8); aneurysm of unspecified site (I72.9); other aneurysm (442); aneurysm of other specified artery (442.8). |
| Secondary outcome | All-cause mortality | Deceased; R99 Ill-defined and unknown cause of mortality |
| Positive control outcome | Secondary hyperparathyroidism | E21.1 Secondary hyperparathyroidism, not elsewhere classified |
| Negative control outcome | Appendicitis / diseases of appendix | K35–K38 Diseases of appendix |

**Supplementary Table 4. Availability of key structured baseline variables before propensity score matching**

| **Variables** | Availability, VDD | Availability, control | Missing, VDD | Missing, control |
| --- | --- | --- | --- | --- |
| eGFR | 80.7% | 73.0% | 19.3% | 27.1% |
| Hemoglobin | 76.8% | 66.9% | 23.2% | 33.1% |
| Albumin | 65.8% | 60.4% | 34.2% | 39.6% |
| BMI | 60.8% | 64.6% | 39.2% | 35.4% |
| Hemoglobin A1c | 41.8% | 33.2% | 58.2% | 66.8% |

Values indicate the proportion of patients with available or missing structured measurements in the TriNetX platform before propensity score matching.
